# Transcriptional Response of Bovine Monocyte-Derived Macrophages after the Infection with Different Argentinean *Mycobacterium bovis* Isolates

**DOI:** 10.1155/2013/458278

**Published:** 2013-01-01

**Authors:** Karina Caimi, Federico Blanco, Marcelo Soria, Fabiana Bigi

**Affiliations:** ^1^Biotechnology Institute, INTA Castelar, N. Repetto y Los Reseros s/n (1686), 1712 Hurlingham, Buenos Aires, Argentina; ^2^Agricultural Microbiology, College of Agronomy, University of Buenos Aires, INBA-CONICET, Avenue San Martín 4453, C1417DSE Buenos Aires, Argentina

## Abstract

Infection of bovines with *Mycobacterium bovis* causes important financial hardship in many countries presenting also a risk for humans. *M. bovis* is known to be adapted to survive and thrive within the intramacrophage environment. In spite of its relevance, at present the information about macrophage expression patterns is scarce, particularly regarding the bovine host. In this study, transcriptomic analysis was used to detect genes differentially expressed in macrophages derived from peripheral blood mononuclear cells at early stages of infection with two Argentinean strains of *M. bovis*, a virulent and an attenuated strains. The results showed that the number of differentially expressed genes in the cells infected with the virulent strain (5) was significantly lower than those in the cells infected with the attenuated strain (172). Several genes were more strongly expressed in infected macrophages. Among them, we detected encoding transcription factors, anthrax toxin receptor, cell division and apoptosis regulator, ankyrin proteins, cytoskeleton proteins, protein of cell differentiation, and regulators of endocytic traffic of membrane. Quantitative real-time PCR of a selected group of differentially expressed genes confirmed the microarrays results. Altogether, the present results contribute to understanding the mechanisms involved in the early interaction of *M. bovis* with the bovine macrophage.

## 1. Introduction

*Mycobacterium bovis* is an important aerobic pathogenic bacterium and the causative agent of most cases of tuberculosis in bovines (BTB). *M. bovis *also poses a zoonotic risk to herd keepers and communities that have close interactions with infected livestock [1]. The impacts of BTB infection are manifold: not only the losses due to the depopulation of herds and the social problems of removing cattle from small holdings, but also the vast economic consequences, particularly in the developing countries, that result from herd restrictions, disruptions to trade, and reduced agricultural productivity [2]. 

The pathogenesis of BTB results from a complex interaction between the pathogen and the host [3]. The gateway of the mycobacteria into the host is through macrophages, where the bacteria can survive a long period of time without the appearance of any symptoms [4]. In response to factors that are still unknown, the population of bacteria begins replication and escapes the infected macrophages causing illness in a small proportion of individuals. The macrophage plays a key role as an effector cell, activating innate immune responses and initiating acquired immune response. Innate immunity provides an important early defense against *M. bovis* and is critical in determining the clinical outcome. In order to survive the immune response, mycobacteria have developed a variety of mechanisms that prevent them from being killed by their host cells [4]. In addition, the level of pathogenicity of mycobacterial species in different hosts can be related to their effects on the host's immune response. It has been observed that the efficiency of phagolysosomal fusion and the rate of bacteria proliferation in macrophages vary among different *M. tuberculosis *clinical isolates [5]. Furthermore, the initial interaction between macrophages and mycobacteria is thought to play a key role in determining the outcome of infection [4].

Large-scale transcriptional gene expression analysis has been used to define the repertoire of genes expressed in host-pathogen interactions of tuberculosis disease and many other infectious diseases [4]. These transcriptional approaches have significantly contributed to understanding the mechanisms involved in these interactions. Interactions of pathogenic *Mycobacterium* species with macrophages from different hosts have been assessed using gene expression profiles [6–10]; however, there is small number of studies where the early responses of bovine macrophages to *M. bovis *have been previously evaluated [11].

For this reason, the aim of the present study was to compare the early gene expression profile of bovine monocyte-derived macrophages (MDMs) obtained from peripheral blood mononuclear cells (PBMCs) following infection with either an attenuated or a hypervirulent *M. bovis* strain. These distinct *M. bovi*s strains were previously studied according to their virulence and replication rate in murine and bovine macrophages [12, 13]. As a result, it has been found that 464 genes were significantly differentially expressed during the early cellular events of macrophage infection. Analysis of the data sheds light on the crosstalk between intracellular pathogens and their host cells, highlighting possible novel mechanisms of bacterial evasion from immunological elimination.

## 2. Materials and Methods

### 2.1. Bacterial Strains and Culture Media

The *M. bovis *strains used in this work were isolated from an Argentinean wild boar (04–303) and from an Argentinean bovine (04–534), respectively. In a previous study by Aguilar and colleagues [12] based on a mouse model of progressive pulmonary tuberculosis, *M. bovis *strain 04–534 showed an attenuated phenotype, whereas strain 04–303 was the most virulent in mice. Therefore, these strains were selected since they represent the extremes in terms of virulence among the ones studied. In addition, the replication rate of strain 04–303 in bovine macrophages has been shown to be significantly higher than that of the strain 04–534, as was described by Blanco and colleagues [13].

Strains were grown in either Middlebrook 7H9 medium or Middlebrook 7H11 medium, both supplemented with albumin/dextrose/catalase, 0.4% pyruvate, and 0.05% Tween 80 to avoid the formation of clumps. Culturing of all *M. bovis* strains was performed in a biosafety containment level (CL3) laboratory. 

### 2.2. PBMCs Isolation and MDMs Infection

The animals used in this study were selected from the experimental herd of INTA and were tested negative for bovine tuberculosis infection for the single intradermal tuberculin test. Six hundred microliters of blood were taken in sterile conditions according the instructions of the Committee for Institutional Care and Use of Animal Experimentation (CICUAE) of INTA. PBMCs were separated from heparinized blood by centrifugation over histopaque 1077 (Sigma, USA) following manufacturer's protocol. To derive monocytes, PBMCs were seeded in T75 flasks (2 × 10^8^) containing RPMI1640 complete medium (Invitrogen, USA) supplemented with 10% of autologous plasma for 16 h at 37°C, 5% CO_2_. Nonadherent cells were removed by washing with Phosphate-buffered saline (PBS). Only adherent cells were maintained in culture for 5 days at 37°C, 5% CO_2_ to obtain MDMs [14]. Cell viability was confirmed by trypan blue exclusion assay [15]. The *M. bovis* cultures were harvested, washed to eliminate rest of bacteria culture medium, suspended in RPMI medium, vortexed, sonicated for 1 min in an ultrasonic cleaner, and passed through a syringe needle (25 gauge) to disaggregate bacteria clumps. Viable bacteria were counted with Live/Dead BacLight Bacterial Viability Kit (Molecular probes, Invitrogen) and then used to infect the cultured bovine macrophages. The infection was performed at a multiplicity of infection (moi) of 5 bacteria/cell. Infected cells were incubated at 37°C, 5% CO_2_ for 4 h and then washed three times with fresh RPMI 1640 medium to eliminate extracellular bacteria. Two independent sets of RNA samples were prepared; one was for the microarrays and the other for the reverse transcription quantitative real-time polymerase chain reaction (RT-qPCR) analysis. Three infections were performed for each set.

### 2.3. Isolation of RNA from Macrophages

Four hours after infection, the cells were scraped and lysed with 1 mL of chilled Trizol (Invitrogen, Carlsbad, USA). Trizol was removed from the flasks and the lysate homogenized. Cellular RNAs extractions were then performed according to the manufacter's instructions for Trizol reagent (Invitrogen, USA). RNA pellets were suspended in 30 *μ*L RNase-free water. RNA samples were further purified using RNeasy Mini Elute Cleanup Kit (Qiagen, Melbourne, Australia) eluted from the column in 20 *μ*L RNase-free water. The RNA quality and integrity was checked before hybridizations using a Bioanalyser 2100 (Agilent).

### 2.4. Microarrays Hybridization

Ten microarray slides were processed in total, representing the biological replicates per group: three for the cell infected with the *M. bovis* virulent strain (04–303), three for cells infected with the *M. bovis* attenuated strain (04–534), and four for the noninfected cells. Affymetrix GeneChip Bovine Genome Array platform (Affymetrix, Santa Clara, USA) was used in this study. The array contains 24,027 probe sets representing over 23,000 transcripts from *Bos taurus* and include approximately 19,000 annotated UniGene clusters. The experiment was designed to be compliant with Minimum Information About a Microarray Experiment (MIAME) standards. Each RNA sample was processed and hybridized to individual slides. Target preparation including verification of RNA quality assessed using a Bioanalyser 2100 (Agilent Technologies, Santa Clara, USA) and microarray processing procedures were carried out at the Affymetrix facility at the School of Agronomy of the University of Buenos Aires, as described in the Affymetrix GeneChip Expression Analysis Manual (Affymetrix), and scanning was performed with a Microarray Scanner 3000 7G (Affymetrix).

### 2.5. Statistical Analysis of Microarrays Data

The analysis of expression data was performed with different packages from the Bioconductor project (http://www.bioconductor.org/) [15], an extension for bioinformatics of the *R* statistical language (http://www.r-project.org/) [16]. The data quality of ten CEL files obtained from the Affymetrix chips were assessed with packages affyPLM [17] and simple affy. On the basis of the boxplots of normalized unscaled standard errors, relative log expressions, and the clustering of between array distances (measured by median absolute deviations), one chip of each treatment was removed from the analysis. The remaining good-quality data was normalized using function rma of the package Affy [18], which essentially reproduces the standardization procedure of the Affymetrix MicroArray Suite (MAS) software. 

To filter the arrays, the genefilter package was used: first those probesets with low variance across samples were removed, and then probesets with little variation between treatments or high levels of noise were filtered out using an Anova filter with a relaxed cutoff of *P* = 0.20. The detection of probesets with differential expression between macrophages infected with virulent or attenuated strains and the uninfected macrophage control was performed with linear models and empirical Bayes methods to correct for multiple comparisons as implemented in the Linear Models for Microarray Data (LIMMA) package [19]. The false discovery rate was set at *P* = 0.05. All microarray data were MIAME compliant and have been submitted to the NCBI Gene Expression Omnibus (GEO) database with experiment series accession number GSE39819.

A functional clustering of lists of differentially expressed probesets was carried out with the Database for Annotation, Visualization, and Integrated Discovery (DAVID) web tool http://david.abcc.ncifcrf.gov/ [20]. The GO annotations were downloaded from the Blast2GO-Functional Annotation Repository (B2G-FAR, http://www.b2gfar.org/).

### 2.6. Validation of Microarrays Results by RT-qPCR

The validation procedure was performed using PBMCs collected (three times) from different animals from those sampled for microarrays experiments. The cells were infected as mentioned above. 

DNA-free RNA (1 *μ*g) was mixed with 50 ng of random primers (Invitrogen) in 20 *μ*L of final volume and reverse transcribed to total cDNA with SuperScript II reverse transcriptase (Invitrogen, USA) following the manufacturer's instructions. One microliter of the cDNA was used as template for each real time quantitative PCR (qPCR) reaction.

All primers were designed using Primer Express Software to span an intron-exon boundary and to anneal only to cDNA synthesized from spliced mRNAs. Primer sequences are listed in Table 1.

qPCR reactions were performed as described by Blanco and colleagues [13]. Briefly, SYBR green QuantiTec Mastermix (Qiagen) was employed, and reactions were made on Applied Biosystem 7000 SDS using standard cycling conditions. All reactions were performed in duplicate, and qPCR data were analyzed using the 2ddCT with efficiency correction as described previously [21] to assess differences on gene expression in macrophages within groups, RNA polymerase II (RPII) gene was used as the control gene, and data from control group was used as the calibrator [22]. Relative Expression Software Tool (REST) beta 9 software (http://www.gene-quantification.de/rest-2009.html) was used for final calculations and statistical analysis [21].

## 3. Results

### 3.1. DNA Microarray Analysis

Bovine monocyte-derived macrophages were infected with either a virulent (Argentinean wild boar 04–303) or an attenuated *M. bovis* strain (Argentinean bovine 04–534) for 4 hours, and cellular transcriptomes were analyzed and compared to the reference transcriptome of uninfected cells. 

Comprehensive gene expression profiles for the three attenuated and the three virulent *M. bovis* macrophage infections were generated with high-density oligonucleotide bovine arrays (Affymetrix) and 24,072 probe sets, which in total interrogated the expression levels of approximately 23,000 transcripts. After the 2-step filtering, 464 probesets were retained. In order to assess the statistical significance of the expression profiles for the 464 probesets obtained in the previous step, LIMMA's linear models and empirical Bayes corrections for multiple comparisons were applied. The number of probe sets with a statistically significant fold change was highly influenced by the filtering and the statistical testing settings. Considering a threshold of 0.05 for the adjusted *P* value, 310 unique probesets showed a differential expression that was statistically significant (see Supplementary Material S1 available online at http://dx.doi.org/10.1155/2013/458278). The results showed that the number of upregulated genes (161) when MDMs were infected with both strains versus control was significantly higher than the number of downregulated genes (15). Furthermore, the number of differentially expressed genes in the cells infected with the virulent strain (5) was significantly less than those in the cells infected with the attenuated strain (172). In Table 2, these results are summarized. 

### 3.2. Description of Highly Differentially Expressed Genes

Among the five exclusively differential expressed genes in the cells infected with the virulent strain (04–303), three were upregulated genes (Table 3 and Supplementary Material S1). One is the interferon-gamma-inducing factor, IL18. IL-18 together with IL-12 cytokines is primarily produced by dendritic cells and macrophages in response to Toll-like receptor (TLR) signalling interaction with tubercle bacilli [23]. Another upregulated gene is the ankyrin repeat domain 17, ANKRD17. The ankyrin superfamily is composed of proteins that are ubiquitously expressed and typically found within the membrane-associated cytoskeleton. Ankyrins contain repeat domains that interact with other ankyrin repeats. These ankyrin interactions act in many diverse functions and include regulation of transcription, cell cycle, cell fate determination, cytoskeletal integrity, cellular mechanosensation, and endocytosis [24, 25]. TCDD-inducible poly (ADP-ribose) polymerase, TiPARP, is the last upregulated gene in this group. Poly(ADP-ribose) polymerases catalyse the covalent attachment of ADP-ribose units from NAD+ to itself and to a limited number of other DNA binding proteins, which decreases their affinity for DNA. Poly(ADP-ribose) polymerase is a regulatory component induced by DNA damage. 2,3,7,8-Tetrachlorodibenzo-p-dioxin (TCDD) causes pleiotropic effects in mammalian species through modulating gene expression. The gene TiPARP is a target of TCDD that may contribute to multiple responses to TCDD by modulating protein function through poly ADP-ribosylation [26].

Genes exclusively upregulated in the cells infected with the attenuated strain (04–534) are involved in different cellular processes (Table 3). DNA transcription and RNA metabolism are some one of these processes and some representative genes that include DNA binding genes, and RNA metabolism genes or transcription factors are present in this group: TAR DNA binding protein (TARDBP); RNA binding motif protein 18 (RBM18); Rho-associated, coiled-coil containing protein kinase 2 (ROCK1); and the gene CREB/ATF bZIP transcription factor (CREBZF) which is a member of the CREB/ATF family of transcription factors that play an important role in cellular growth, metabolism, and survival [27].

The gene clathrin interactor 1 (CLINT1) was found also upregulated in this group. It was reported that CLINT1 might participate in the formation of clathrin-coated vesicles at the level of the trans-Golgi network (TGN). Clathrin-coated vesicles participate in receptor-mediated endocytosis, in the transport of lysosomal enzymes from the TGN to the endosomes/lysosomes, and in the sorting of receptors within the endosomal system [28].

Another important overexpressed gene is the interferon alpha receptor 1 (IFNAR1). Although type I interferon is key to the immune response to viral pathogens, its role in bacterial infections is less understood. Recently mice lacking this receptor (IFNAR^−/−^) have enhanced their resistance to infection with* Listeria monocytogenes,* an intracellular pathogen like *Mycobacterium* sp. [29]. Importantly, upregulation of type-1 interferon (*α*/*β*) mediated signalling has been recently described during active TB disease in patients and also in the lungs of *M. tuberculosis* infected mice and in infected human macrophages [30].

The group of genes that was significantly upregulated (fold change >2) both in the macrophage infected with virulent and attenuated *M. bovis *strains, encode proteins involved in transcription process, in intracellular trafficking, and in the regulation of cell processes. Established functions have been previously assigned for several of these differentially expressed genes (Table 3).

Anthrax toxin receptor 2 (ANTXR2) is one of the genes up-regulated in both macrophage infections. It has been demonstrated that ANTXR2 is expressed in primary mononuclear phagocytes and facilitates the entry of *Bacillus anthracis* exotoxins into the cytosol of susceptible cells [31].

Other genes upregulated in both macrophage infections were myotrophin (MPTN) the EH domain containing 4 (EHD4). MPTN is another member of the ankyrin superfamily previously mentioned. Members of the EHD protein family (EHD1–4) have emerged as critical regulators of endocytic membrane traffic as well as cell surface receptors [32]. Remarkably, an EH domain has been found upregulated in human alveolar macrophages infected with *M. tuberculosis *H37Rv [8].

Genes encoding regulator proteins or intracellular trafficking proteins were also found differentially expressed. Such are the examples of the nuclear receptor corepressor 1 (NCOR) which has a role in the regulation of inflammatory response genes in macrophages [33] and the kinesin family member 3 (KIF3A) which regulates the vesicular transport in leucocytes and monocytes [34]. It has been reported that kinesins KIF5B and KIF5C are directly associated with RanBP2, a zinc finger, and RAN binding domain containing protein [35]. Interestingly, in the present work it was found that together with KIF3A, a zinc finger RAN binding domain containing 2 (ZRANB2) was also upregulated in macrophages infected with both *M. bovis* strains. Further studies are necessary to demonstrate a functional connection between both proteins in the infected macrophages.

In addition, it has been reported that CARP/CCAR1 induces apoptosis in a manner dependent on phosphorylation of a specific tyrosine residue of CARP1 and downstream activation of p38 MAPK and caspase-3 and -9 in HBC cells [36]. Although this function of CARP1 has not been yet reported in monocytes, it is consistent with the apoptotic activity induced in the macrophage upon infection with some *M. tuberculosis* isolates.

Another overexpressed gene is ZBTB1 that encodes zinc finger and BTB domains. Established functions for zinc finger-BTB domains containing proteins have been reported, such as the promyelocytic leukemia zinc finger protein (PLZF) that induces monocytic differentiation in hematopoietic cells [37].

Other proteins with a probable role in transcription, splicing and different aspects of cellular RNA metabolism whose genes were overexpressed after macrophage infections are LOC515193, similar to SAFB like transcription modulator; a YTH protein family member; RQCD1, a protein required for cell differentiation, and the X-inactive specific transcript (XIST gene) which is the only gene known to be specifically transcribed from the inactive X chromosome in female somatic cells and is necessary and sufficient for the initiation and spread of X inactivation. The role of this last gene in the early cellular events of macrophage infection is unclear.

Although all these differential expressed proteins may play roles in cellular processes induced after mycobacterial infections, understanding the relationship of these protein functions with *M. bovis* infection needs further investigation.

*Gene Set Enrichment Analysis*. The probesets differentially expressed in macrophage cells infected with the virulent and the attenuated strains versus the control cells were submitted to the DAVID web application for their functional clustering. This procedure clusters genes according to their similarities in functional annotations (Supplementary Material S2).

For the virulent versus control contrast, only one cluster with 5 genes was found, and their annotations were related to nucleotide binding and more specifically to ATP binding. A detailed description of the genes included in this cluster is provided below.

For the attenuated versus control contrast, seven clusters were found with DAVID's functional gene clustering.

The first cluster contained 5 probe sets whose common annotations were RNA recognition motif and nucleotide binding. 

The second cluster contained 7 probe sets with proteolysis-related annotations. 

The third cluster contained 5 probe sets enriched in regulators of small GTPases and related annotations, including GTPase-activating proteins (GAP) and guanidine exchange factor (GEF). GAPs are a family of regulatory proteins whose members can bind and activate small GTPases and others G proteins. Regulation of small GTPases is important because they participate in crucial cellular processes like cellular trafficking and cell cycling. A representative of this cluster is RASA2 that encodes a member of the GAP1 family. This GAP stimulates the GTPase activity of normal RAS p21. In this cluster, a gene containing a TBC domain and IQGAP1 is also present. IQGAP1 is a protein scaffold that binds to actin filaments and also to Rac and Cdc42, members of the Rho family of small GTPases [38], while TBC domains have been expected to function as a certain Rab-GAP [39]. On the other hand, the function of GEF proteins is to activate Rho GTPases, which are important regulators of multiple cellular activities and, most notably, reorganization of the actin cytoskeleton [40]. FGDA, a gene also present in cluster three, encodes a GEF protein, which activates Cdc42 that regulates a variety of cellular functions including cell shape, migration, endocytosis, and cell cycle.

The fourth cluster contained 17 probesets, and 3 of them were also part of the only cluster found for the virulent strain versus control. In addition to the nucleotide binding and ATP binding, there were other enriched annotations, like protein kinase and phosphorylation. Genes encoding members of mitogen-activated protein kinases (MAPKs) and cyclin-dependent kinases (CDKs) are present in this cluster. Kinases are known to regulate the majority of cellular pathways, especially those involved in signal transduction. In particular, MAP kinases are mainly involved in mechanisms of immune responses, from innate immunity to activation of adaptive immunity and to cell death, while CDKs (PRPF4B and CDK2) form a family of protein kinases mostly involved in regulating the cell cycle. Other groups of kinases included in the cluster are ToR (TOR1B) that participates in signaling pathways of autophagy [41] and tribbles (TRIB3) that regulate MAPKs [42].

The fifth cluster contained 4 probe sets, all with annotations “purine nucleoside triphosphate biosynthetic process” and similar. Three of these clustered genes encode ATPases, one V-ATPase and two P-type cation transport ATPases that are involved in acidification of intracellular compartments and calcium homeostasis. The V-ATPase is a multisubunit enzyme that mediates acidification of eukaryotic intracellular organelles, and the role of this enzyme in the antimicrobial mechanism against pathogenic mycobacteria has been established. The P-type ATPases remove bivalent calcium ions from eukaryotic cells against very large concentration gradients and play a critical role in intracellular calcium homeostasis [43]. Calcium homeostasis participates in apoptosis in different systems and regulates important cellular events triggered upon infection of macrophages with pathogenic mycobacteria [44] in turn; mycobacteria have developed mechanisms to evade these antimicrobial responses [45, 46].

The sixth cluster had 4 probesets include transmembrane and transport involved domains. 

Finally, the seventh cluster included 4 probesets with “zinc binding annotations” and zinc fingers domains. 

**Figure 1:**
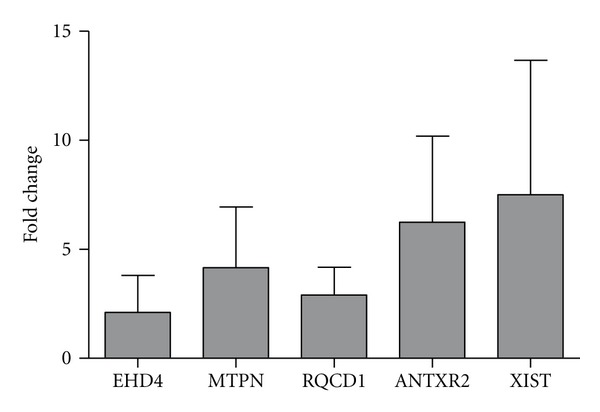
Gene expression fold-change differences between PBMC from *M. bovis *303 and 534 infected cells and control uninfected cells using RT-qPCR. Relative gene expression was calculated using the 2-DDCt method with *E* correction, using RNA *pol *II mRNA expression as reference gene and the uninfected cells condition as the calibrator. The bars indicate the average ratios of infected cells/uninfected cells ±SEM.

*Validation of Microarray Analysis by RT-qPCR*. The microarray data for the genes whose expression varied at least two fold between conditions were validated for a subset of five genes by RT-qPCR. For this purpose, PBMCs were collected (three times) from different animals to those sampled for the microarrays experiments. The RT-qPCR results corroborated the microarray data for all genes tested. As shown in Figure 1, the fold changes determined by RT-qPCR were often greater than the fold changes for the same genes determined by microarray analysis. Thus, the concordance between the microarray results and the RT-qPCR results was complete, supporting the statistical approach that was adopted in this study.

## 4. Discussion

In the present study, the early (four hours) gene expression profile induced in bovine blood MDMs after infection with two *M. bovis* strains with distinct level of virulence was compared to that of uninfected macrophages [12]. PBMCs represent an accessible tissue for the development of improved diagnostics, and previous studies have shown that immune responses generated in the peripheral blood of bovines infected with tuberculosis reflect those at the site of disease [47].

Comparative gene expression profile of virulent strain-infected macrophages and attenuated strain-infected macrophages did not identify significant differences suggesting that during the early events of intracellular replication both strains have equivalent fitness in spite of their differences in virulence [12] and in replication rate both in mice and bovine macrophages [13]. In agreement with this, a microarray analysis study on bovine alveolar macrophages infected with a virulent and an attenuated isogenic *M. bovis* strains has detected small differences in gene expression between both strains and has only identified IL-8 gene as significantly overexpressed in macrophages infected with the virulent strain [8]. As well, other differential expressed genes identified in this microarray analysis study were not confirmed as significantly different in RT-qPCR validations [8]. Unexpectedly, in the work presented here, the expression of IL-8 did not show a significant difference among the condition analyzed, suggesting differences in both infection models studied: alveolar and monocyte.

The present work shows that the expression of few genes was considerably changed after *M. bovis* infection, irrespectively of the strain used to infect the macrophages. However, at least five genes were highly upregulated in macrophages infected with the virulent *M. bovis* strains, which gives strength to the results obtained here.

As this study was limited to the analysis of *M. bovis*-macrophage interactions, the possibility that the differentially expressed genes here identified were part of a shared macrophage activation program induced by intracellular bacteria cannot be discarded. In this regards, Nau and colleagues have performed a comparative examination of the transcriptional responses of human macrophages to a variety of bacteria, including *M. tuberculosis *[48]. The authors have found that macrophages responded to bacteria with a robust, shared pattern of gene expression, encoding receptors, signal transduction molecules, and transcription factors. Remarkably, the set of differentially expressed genes identified by these authors did not include any of the highly upregulated genes identified in the present study, suggesting that there are differences in response according to different species, with bovine macrophages responding differently than human macrophages.

A previous global transcriptional profile analysis between macrophages and dendritic cells in humans has revealed that the expression of more than two thousand genes was altered during 48-hour infection with *M. tuberculosis. *This altered gene expression profile included genes involved in sensing pathogens, intracellular signalling and trafficking, cell motility, and cytoskeleton remodelling, presenting profound differences between cell type in genes involved in oxidative stress, intracellular vesicle acidification and trafficking [7]. In concordance with these previous findings, the present study found that differential expressed genes were clustered in functional annotations involved in different aspects of phagocytosis, such as cellular trafficking, endocytocis, cell migration, morphology, and cell cycling. Furthermore, it was found that the most significantly upregulated genes (fold change >4) in macrophages infected with *M. bovis* include those involved in DNA interaction, nuclear receptor, vesicular transport, and apoptosis regulation.

Silver and colleagues have reported that cytokines IL-23, IL-6, and TNF-*α* were significantly upregulated in human alveolar macrophages infected during 24 hours with H37Rv, while IL-12 was not [9]. In the present study, overexpression of these cytokines was not observed, and the reason for this discrepancy could be the different origin of the macrophages used in both studies. In fact, in the study by Silver and colleagues, it is shown that IL-23 is not produced in blood monocytes [9]. Remarkably, it was found in the present work that among the upregulated genes in macrophages infected with the virulent *M. bovis* strains was that encoding IL-18. IL-18 together with IL-12 cytokines, is primarily produced by dendritic cells and macrophages in response to Toll-like receptor (TLR) signalling interaction with tubercle bacilli [21]. In addition, both *M. bovis* strains induced the upregulation of IL-13 receptor. It has been demonstrated that IL-13 can subvert Th1-mediated immunity and promote inappropriate activation of macrophages, abrogating IFN-*γ* induced autophagy-mediated killing of intracellular mycobacteria in murine and human macrophages [49]. One possible reason for the lack of other cytokine expression in our studies is the short period of infection. It has been reported that the peak of cytokine productions in bovine macrophages is 24 hours after *M. bovis* infections [8].

Altogether, the present results contribute to define the transcriptional responses induced during the early events that occur in *M. bovis* macrophage interactions. However, it is important to take into consideration that some of the gene expression changes observed in this study may not be specific for *M. bovis* infection and may represent a shared transcriptional program induced by infectious diseases. Thus, further studies are needed to investigate this possibility.

## Supplementary Material

S1. The list of differentially expressed bovine macrophage transcripts detected post infection with M. bovis virulent and attenuated strain vs control uninfected cells.
S2. Functional clustering of differentially expressed bovine macrophage transcripts. The probesets differentially expressed in macrophage cells infected with the virulent and the attenuated strains versus the control cells were submitted to the DAVID web application for their functional clustering. The top ranking functional categories identified by DAVID are listed according to P-values. Cells infected with the virulent strain vs control cells rendered one functional cluster. Cells infected with the attenuated strain vs. control cells rendered 7 different clusters.

## Figures and Tables

**Table 1:** Primers sequences used for RT-qPCR.

Genes	Forwardprimer (5′–3′)	Reverse primer (5′–3′)
EDH4	ACCAAGTTCCACTCGCTGAA	GCTCATCTCCTCCTGGCTAA
MPTN	TTGAAGCCACTGACAACCAG	AACAGACAGACAGGCAGCAG
RQCD1	GCCAAAGGACAGCAGGAAC	CAGCGAAAGCCATAAGCAA
ANTXR2	CCTCCTCCACCACCACCT	TTATCACCCCAACGAACCTC
XIST	GGGGTGTTTGACCGTTACAT	TCCCTCCTTTCACTTTGTCC
RPII	GCACCACGTCCAATGACAT	GTGCGGCTGCTTCCATAA

**Table 2:** Analysis of probesets with differential expression.

Contrast	Upregulated	Downregulated	Total
04–303 versus control	3 (3)*	2 (2)	5 (5)
04–534 versus control	159 (114)	13 (13)	172 (127)
04–303 versus control and 04–534 versus control	125 (92)	8 (5)	133 (97)

*Numbers in parenthesis are the number of probesets that were mapped to an NCBI gene database (http://www.ncbi.nlm.nih.gov/gene).

**Table 3:** Genes upregulated in PBMCs of *M. bovis* infected and control cells.

Probeset ID	Gene symbol	Gene entrez ID	Gene name	Log FC*	FC	*P* value	*P*_adj._ value
Probesets differentially expressed in 303 infected versus control (cut-off: *P*_adj._≤ 0.05)							

Bt.28345.1.S1_at	ANKRD17	508405	Ankyrin repeat domain 17	1,456	2,743	6,628*E* − 03	0,032783
Bt.234.1.S1_at	IL18	281249	Interleukin 18 (interferon-gamma-inducing factor)	1,440	2,714	7,235*E* − 03	0,033570
Bt.23611.3.S1_at	TIPARP	540975	TCDD-inducible poly(ADP-ribose) polymerase	1,340	2,531	1,245*E* − 02	0,042252

Probesets differentially expressed in 303 infected versus control and 534 infected versus control (cut-off: *P*_adj._≤ 0.05)							

Bt.19496.1.A1_at	ND**	ND	ND	2,753	6,743	3,035*E* − 07	0,000141
Bt.23911.1.A1_at	XIST	338325	X (inactive)-specific transcript	2,603	6,078	1,270*E* − 06	0,000295
Bt.28320.1.A1_at	ND	ND	ND	2,528	5,769	2,532*E* − 06	0,000392
Bt.6566.2.S1_a_at	ND	ND	ND	2,279	4,854	2,194*E* − 05	0,002512
Bt.647.1.S1_at	MTPN	541099	Myotrophin	2,194	4,575	4,399*E* − 05	0,002707
Bt.11778.1.A1_at	ND	ND	ND	2,178	4,526	4,974*E* − 05	0,002707
Bt.18321.1.A1_at	GNB4	525962	Guanine nucleotide binding protein (G protein), beta polypeptide 4	2,158	4,464	5,823*E* − 05	0,002707
Bt.23678.1.A1_at	RQCD1	536537	RCD1 required for cell differentiation1 homolog (*S. pombe*)	2,158	4,463	5,834*E* − 05	0,002707
Bt.12745.3.S1_at	ANTXR2	510080	Anthrax toxin receptor 2	2,093	4,266	9,681*E* − 05	0,004084
Bt.2096.2.S1_at	EHD4	505206	EH domain containing 4	2,070	4,200	1,150*E* − 04	0,004446

Probesets differentially expressed in 534 infected versus control (cut-off: *P*_adj._≤ 0.05)							

Bt.24940.1.A1_at	ND	ND	ND	2,762	6,784	2,698*E* − 06	1,043*E* − 04
Bt.5395.1.S1_a_at	VCAN	282662	Versican	2,116	4,336	3,182*E* − 04	2,636*E* − 03
Bt.24975.1.S1_at	YTHDC2	541024	YTH domain containing 2	2,037	4,104	5,290*E* − 04	3,880*E* − 03

*FC: fold change; **ND: not defined.
