# Supplementary material for: Transcriptional Response of Bovine Monocyte-Derived Macrophages after the Infection with Different Argentinean Mycobacterium bovis Isolates
Source: Biomed Res Int. 2013 Jan 1;2013:458278. doi: 10.1155/2013/458278 (PMC3581155; doi:10.1155/2013/458278)
Supplement: Supplementary file 1 — S1. The list of differentially expressed bovine macrophage transcripts detected post infection with M. bovis virulent and attenuated strain vs control uninfected cells. S2. Functional clustering of differentially expressed bovine macrophage transcripts. The probesets differentially expressed in macrophage cells infected with the virulent and the attenuated strains versus the control cells were submitted to the DAVID web application for their functional clustering. The top ranking functional categories identified by DAVID are listed according to P-values. Cells infected with the virulent strain vs control cells rendered one functional cluster. Cells infected with the attenuated strain vs. control cells rendered 7 different clusters. [file 458278.f1.pdf]

Supplementary Material - S1 -

Probesets differentially expressed in 303 infected vs. control (cut-off: p.adj <= 0.05)

probeset ID	Gene symbol	Gene Entrez ID	Gene name	logFC	FC	P.Value	adj.P.Val
Bt.28345.1.S1_at	ANKRD17	508405	ankyrin repeat domain 17	1,456	2,743	6,628E-03	0,032783
Bt.234.1.S1_at	IL18	281249	interleukin 18 (interferon-gamma-inducing factor)	1,440	2,714	7,235E-03	0,033570
Bt.23611.3.S1_at	TIPARP	540975	TCDD-inducible poly(ADP-ribose) polymerase	1,340	2,531	1,245E-02	0,042252
Bt.24217.1.S1_at	NFE2	514006	nuclear factor (erythroid-derived 2), 45kDa	-1,364	-2,575	1,095E-02	0,039242
Bt.12504.1.S1_at	CLU	280750	clusterin	-1,670	-3,182	1,852E-03	0,016635

Probesets differentially expressed in 303-infected vs. control and 534-infected vs. control (cut-off: p.adj <= 0.05)

probeset ID	Gene symbol	Gene Entrez ID	Gene name	logFC	FC	P.Value	adj.P.Val
Bt.19496.1.A1_at				2,753	6,743	3,035E-07	0,000141
Bt.23911.1.A1_at	XIST	338325	X (inactive)-specific transcript	2,603	6,078	1,270E-06	0,000295
Bt.28320.1.A1_at				2,528	5,769	2,532E-06	0,000392
Bt.6566.2.S1_a_at				2,279	4,854	2,194E-05	0,002512
Bt.647.1.S1_at	MTPN	541099	myotrophin	2,194	4,575	4,399E-05	0,002707
Bt.11778.1.A1_at				2,178	4,526	4,974E-05	0,002707
Bt.18321.1.A1_at	GNB4	525962	guanine nucleotide binding protein (G protein), beta polypeptide 4	2,158	4,464	5,823E-05	0,002707
Bt.23678.1.A1_at	RQCD1	536537	RCD1 required for cell differentiation1 homolog (S. pombe)	2,158	4,463	5,834E-05	0,002707
Bt.12745.3.S1_at	ANTXR2	510080	anthrax toxin receptor 2	2,093	4,266	9,681E-05	0,004084
Bt.2096.2.S1_at	EHD4	505206	EH-domain containing 4	2,070	4,200	1,150E-04	0,004446
Bt.17932.2.S1_a_at	CCNL1	514613	cyclin L1	2,017	4,048	1,712E-04	0,005775
Bt.20947.2.S1_at	LOC100140708	100140708	similar to IQ motif containing GTPase activating protein 2	2,015	4,041	1,742E-04	0,005775
Bt.22594.1.S1_at	LOC789894	789894	similar to PAP-associated domain-containing protein 5 (Topoisomerase-related function protein 4-2) (TRF4-2)	1,959	3,887	2,630E-04	0,007072
Bt.27386.1.A1_at	ANKRD50	527956	ankyrin repeat domain 50	1,941	3,840	2,983E-04	0,007284
Bt.18060.1.A1_at				1,922	3,789	3,424E-04	0,007943
Bt.1699.2.S1_a_at	RBPJ	767928	similar to Recombining binding protein suppressor of hairless (J kappa-recombination signal-binding protein) (RBP-J kappa) (RBP-J) (RBP-JK) (CBF-1) (Renal carcinoma antigen NY-REN-30); recombination signal binding protein for immunoglobulin kappa J region	1,884	3,691	4,465E-04	0,008711
Bt.11954.1.S1_at	SLTM	515193	similar to SAFB-like transcription modulator (Modulator of estrogen-induced transcription)	1,883	3,688	4,505E-04	0,008711
Bt.13284.1.A1_at				1,882	3,687	4,516E-04	0,008711
Bt.2663.2.S1_at	KRAS	541140	v-Ki-ras2 Kirsten rat sarcoma viral oncogene homolog	1,880	3,681	4,594E-04	0,008711
Bt.7131.2.S1_at	PLDN	614408	pallidin homolog (mouse)	1,874	3,666	4,785E-04	0,008711
Bt.15806.1.A1_at	IREB2	539404	similar to iron-responsive element binding protein 2	1,868	3,649	5,010E-04	0,008711

Bt.24258.2.S1_at	MAN1A1	530027	mannosidase, alpha, class 1A, member 1	1,866	3,645	5,069E-04	0,008711
Bt.9710.1.S1_at	PRPF4B	512236	PRP4 pre-mRNA processing factor 4 homolog B (yeast)	1,840	3,580	6,057E-04	0,009319
Bt.20931.1.S1_at	PPP1CB	538829	protein phosphatase 1, catalytic subunit, beta isoform	1,836	3,570	6,226E-04	0,009319
Bt.22109.1.S1_at	GOPC	541186	golgi-associated PDZ and coiled-coil motif containing	1,824	3,541	6,747E-04	0,009693
Bt.9438.1.S1_at	SYNCRIP	511763	synaptotagmin binding, cytoplasmic RNA interacting protein	1,813	3,513	7,291E-04	0,009693
Bt.3311.3.S1_at				1,812	3,512	7,312E-04	0,009693
Bt.24221.2.S1_at				1,780	3,434	9,091E-04	0,011558
Bt.25292.1.A1_at				1,776	3,426	9,295E-04	0,011558
Bt.7672.1.A1_at				1,774	3,419	9,466E-04	0,011558
Bt.15677.1.A1_at	LOC100299693	100299693	PRP38 pre-mRNA processing factor 38 (yeast) domain containing B	1,763	3,393	1,018E-03	0,011968
Bt.17514.1.S1_at				1,761	3,389	1,032E-03	0,011968
Bt.25051.1.A1_at	TTC14	512635	tetratricopeptide repeat domain 14	1,738	3,336	1,196E-03	0,013538
Bt.16124.2.S1_at				1,733	3,325	1,234E-03	0,013630
Bt.6276.2.S1_at	RAB6A	616537	RAB6A, member RAS oncogene family; RAB6B, member RAS oncogene family	1,724	3,304	1,308E-03	0,014119
Bt.6180.2.S1_at	FRG1	512533	FSHD region gene 1; hypothetical LOC780996	1,717	3,287	1,372E-03	0,014425
Bt.21071.1.S1_at				1,714	3,281	1,399E-03	0,014425
Bt.9847.1.S1_at	C22H3orf63	510095	chromosome 3 open reading frame 63 ortholog	1,708	3,266	1,458E-03	0,014551
Bt.26872.1.S1_at	NIPSNAP3A	534363	nipsnap homolog 3A (C. elegans)	1,705	3,260	1,481E-03	0,014551
Bt.24590.2.S1_at	LSM14A	538838	LSM14A, SCD6 homolog A (S. cerevisiae)	1,703	3,255	1,505E-03	0,014551
Bt.24537.1.A1_at	ZRANB2	508905	zinc finger, RAN-binding domain containing 2	1,690	3,226	1,635E-03	0,015483
Bt.24691.1.A1_at	MLL5	539121	myeloid/lymphoid or mixed-lineage leukemia 5 (trithorax homolog, Drosophila)	1,675	3,193	1,793E-03	0,016635
Bt.9845.1.S1_at	TAPT1	615598	transmembrane anterior posterior transformation 1	1,669	3,180	1,864E-03	0,016635
Bt.22626.2.S1_at	ANKRD12	534739	ankyrin repeat domain 12	1,657	3,154	2,005E-03	0,017432
Bt.2478.2.S1_at	VPS26A	510574	vacuolar protein sorting 26 homolog A (S. pombe)	1,655	3,150	2,029E-03	0,017432
Bt.13706.2.S1_at	COMMD10	538706	COMM domain containing 10	1,651	3,140	2,087E-03	0,017610
Bt.29874.1.S1_at	EIF2C3	406231	eukaryotic translation initiation factor 2C, 3	1,637	3,110	2,275E-03	0,018852
Bt.25965.1.A1_at	NCOR1	507663	nuclear receptor co-repressor 1	1,632	3,100	2,340E-03	0,018864
Bt.1615.1.A1_at	DENND4A	514906	DENN/MADD domain containing 4A	1,631	3,098	2,358E-03	0,018864
Bt.28621.1.S1_at				1,620	3,075	2,519E-03	0,019808
Bt.23342.1.S1_at				1,612	3,056	2,658E-03	0,020221
Bt.19645.1.A1_at	CHD1	505123	chromodomain helicase DNA binding protein 1	1,603	3,037	2,810E-03	0,021029
Bt.17148.2.S1_at	FEM1C	541180	fem-1 homolog c (C. elegans)	1,596	3,022	2,930E-03	0,021320
Bt.20770.1.A1_at				1,591	3,012	3,020E-03	0,021560
Bt.16661.1.A1_at	BAZ1A	540621	bromodomain adjacent to zinc finger domain, 1A	1,582	2,993	3,191E-03	0,022431
Bt.9176.2.S1_at	CUL4B	539668	cullin 4B	1,574	2,976	3,349E-03	0,023195
Bt.6943.1.S1_at				1,564	2,957	3,542E-03	0,024167

Bt.9603.2.S1_at	INTS8	100124522	integrator complex subunit 8	1,561	2,951	3,605E-03	0,024243
Bt.20080.1.S1_at	WSB1	614851	WD repeat and SOCS box-containing 1	1,540	2,908	4,085E-03	0,026693
Bt.7150.2.S1_at	BIRC6	534211	baculoviral IAP repeat-containing 6	1,536	2,900	4,186E-03	0,026933
Bt.18474.2.A1_at	SHOC2	511417	soc-2 suppressor of clear homolog (C. elegans)	1,532	2,892	4,278E-03	0,026933
Bt.2177.2.S1_at	FAM114A1	511583	similar to Family with sequence similarity 114, member A1	1,530	2,887	4,344E-03	0,026933
Bt.6824.1.A1_at	TET2	538481	similar to CXXC finger 6	1,529	2,886	4,353E-03	0,026933
Bt.13614.1.A1_at	ARMC1	514000	armadillo repeat containing 1	1,523	2,874	4,510E-03	0,027186
Bt.22620.1.S1_at	ZBTB1	526903	zinc finger and BTB domain containing 1	1,523	2,874	4,511E-03	0,027186
Bt.24678.1.A1_at	TBC1D9	537817	TBC1 domain family, member 9 (with GRAM domain)	1,520	2,869	4,581E-03	0,027252
Bt.120.1.S1_at	ROCK2	282041	E2F transcription factor 6; Rho-associated, coiled-coil containing protein kinase 2	1,516	2,861	4,694E-03	0,027298
Bt.29953.2.S1_at	CCNYL1	538167	cyclin Y-like 1	1,511	2,851	4,835E-03	0,027694
Bt.22564.2.S1_at	GGTA1	281780	alpha-galactosyltransferase 1 (glycoprotein)	1,496	2,820	5,283E-03	0,029893
Bt.23265.1.S1_at	SPRED1	519882	sprouty-related, EVH1 domain containing 1	1,489	2,807	5,494E-03	0,030409
Bt.9631.1.S1_at	MFN1	515180	similar to mitofusin 1	1,487	2,803	5,560E-03	0,030409
Bt.21982.1.S1_a_at	IQGAP1	532715	IQ motif containing GTPase activating protein 1	1,483	2,796	5,680E-03	0,030409
Bt.17311.2.S1_at	RP2	534054	retinitis pigmentosa 2 (X-linked recessive)	1,481	2,791	5,767E-03	0,030409
Bt.4095.2.S1_at	TOR1B	533928	torsin family 1, member B (torsin B)	1,481	2,791	5,767E-03	0,030409
Bt.17129.2.A1_at				1,472	2,774	6,051E-03	0,031547
Bt.2105.2.A1_at	LATS1	535935	LATS, large tumor suppressor, homolog 1 (Drosophila)	1,460	2,751	6,489E-03	0,032760
Bt.11073.1.S1_at	C8H9orf21	616897	chromosome 9 open reading frame 21 ortholog	1,460	2,750	6,496E-03	0,032760
Bt.16920.1.A1_s_at				1,451	2,733	6,827E-03	0,033345
Bt.13818.1.S1_at	SIRT1	613629	sirtuin (silent mating type information regulation 2 homolog) 1 (S. cerevisiae)	1,442	2,718	7,153E-03	0,033570
Bt.5542.2.S1_at	LOC540136	540136	similar to Nucleosome assembly protein 1-like 1 (NAP-1-related protein) (hNRP); similar to rCG48740; hypothetical protein LOC790872	1,442	2,717	7,173E-03	0,033570
Bt.28750.1.S1_at	IL13RA1	407110	interleukin 13 receptor, alpha 1	1,442	2,716	7,181E-03	0,033570
Bt.28702.1.S1_at				1,440	2,714	7,235E-03	0,033570
Bt.8264.1.A1_at				1,436	2,705	7,423E-03	0,034101
Bt.864.2.S1_at	SRSF1	615796	splicing factor, arginine/serine-rich 1	1,431	2,697	7,601E-03	0,034232
Bt.28189.2.S1_at				1,431	2,696	7,627E-03	0,034232
Bt.24692.1.S1_at	ADRBK2	282136	adrenergic, beta, receptor kinase 2	1,430	2,694	7,673E-03	0,034232
Bt.633.3.S1_at	SFXN1	511375	sideroflexin 1	1,426	2,687	7,841E-03	0,034254
Bt.8353.3.S1_at	HEATR1	506978	HEAT repeat containing 1	1,424	2,683	7,920E-03	0,034254
Bt.26700.1.S1_at	HNMT	613413	histamine N-methyltransferase	1,423	2,681	7,962E-03	0,034254
Bt.9162.1.A1_at	CHORDC1	505144	cysteine and histidine-rich domain (CHORD)-containing 1	1,420	2,676	8,094E-03	0,034254
Bt.18167.1.A1_at				1,420	2,675	8,111E-03	0,034254
Bt.17444.1.S1_at				1,419	2,675	8,120E-03	0,034254

Bt.3964.1.S1_at	SLC2A3	282358	solute carrier family 2 (facilitated glucose transporter), member 3	1,412	2,660	8,481E-03	0,035306
Bt.24447.2.S1_at	F2RL2	512581	coagulation factor II (thrombin) receptor-like 2	1,411	2,659	8,522E-03	0,035306
Bt.11034.1.S1_at	MID1IP1	615572	MID1 interacting protein 1 (gastrulation specific G12 homolog (zebrafish))	1,406	2,651	8,725E-03	0,035603
Bt.2098.2.S1_at	ARHGEF12	503575	Rho guanine nucleotide exchange factor (GEF) 12	1,406	2,650	8,747E-03	0,035603
Bt.1455.1.S1_at				1,400	2,639	9,029E-03	0,036098
Bt.4787.1.S1_at	EEF2K	521730	eukaryotic elongation factor-2 kinase	1,398	2,635	9,152E-03	0,036098
Bt.28133.1.S1_at	MLL3	789336	similar to Myeloid/lymphoid or mixed-lineage leukemia protein 3 homolog (Histone-lysine N-methyltransferase, H3 lysine-4 specific MLL3) (Homologous to ALR protein) (Lysine N-methyltransferase 2C)	1,396	2,631	9,239E-03	0,036098
Bt.25055.1.A1_at	IPMK	615135	inositol polyphosphate multikinase	1,395	2,631	9,262E-03	0,036098
Bt.10153.1.S1_at	EIF2C4	528046	eukaryotic translation initiation factor 2C, 4	1,395	2,629	9,305E-03	0,036098
Bt.21987.2.S1_a_at	PPIG	783720	peptidylprolyl isomerase G (cyclophilin G)	1,394	2,627	9,349E-03	0,036098
Bt.23618.1.A1_at				1,392	2,625	9,413E-03	0,036098
Bt.8169.1.S1_at	SLC39A6	524456	solute carrier family 39 (zinc transporter), member 6	1,380	2,603	1,006E-02	0,037836
Bt.24376.1.S1_at	MTSS1	523498	metastasis suppressor 1	1,379	2,601	1,011E-02	0,037836
Bt.24546.1.S1_at	SMEK1	407220	SMEK homolog 1, suppressor of mek1	1,375	2,595	1,031E-02	0,038279
Bt.19243.2.S1_at	SNX6	532685	similar to sorting nexin 6; sorting nexin 6	1,368	2,581	1,074E-02	0,039242
Bt.27529.1.S1_at	NUP155	506738	nucleoporin 155kDa	1,364	2,574	1,095E-02	0,039242
Bt.27177.1.A1_at	MAP7D3	506950	MAP7 domain containing 3	1,361	2,569	1,112E-02	0,039382
Bt.648.2.S1_at	LRRFIP1	616994	leucine rich repeat (in FLII) interacting protein 1	1,350	2,549	1,182E-02	0,041433
Bt.1711.1.A1_at	LOC615263	615263	hypothetical protein LOC615263	1,349	2,547	1,188E-02	0,041433
Bt.13990.2.S1_at	ZCCHC8	539598	zinc finger, CCHC domain containing 8	1,341	2,533	1,238E-02	0,042252
Bt.3197.1.A1_at	CCAR1	767587	cell division cycle and apoptosis regulator 1	1,341	2,533	1,241E-02	0,042252
Bt.6800.1.A1_at				1,339	2,530	1,249E-02	0,042252
Bt.12606.1.S1_a_at				1,337	2,526	1,265E-02	0,042252
Bt.21348.1.A1_a_at	SNRNP48	513601	small nuclear ribonucleoprotein 48kDa (U11/U12)	1,337	2,526	1,266E-02	0,042252
Bt.13324.1.S1_a_at	IDH1	281235	isocitrate dehydrogenase 1 (NADP+), soluble; similar to Isocitrate dehydrogenase 1 (NADP+), soluble	1,334	2,522	1,282E-02	0,042485
Bt.29945.1.S1_at	PCMTD1	521261	protein-L-isoaspartate (D-aspartate) O-methyltransferase domain containing 1	1,333	2,519	1,293E-02	0,042548
Bt.22090.2.S1_at				1,330	2,514	1,311E-02	0,042849
Bt.22822.2.S1_at				1,327	2,509	1,332E-02	0,043230
Bt.24697.1.A1_at	KIF3A	541246	kinesin family member 3A	1,312	2,482	1,443E-02	0,045863
Bt.19799.1.S1_at	SPAG9	540689	sperm associated antigen 9	1,301	2,464	1,522E-02	0,047738
Bt.6017.2.S1_at				1,301	2,463	1,527E-02	0,047738
Bt.12220.1.S1_a_at				1,300	2,462	1,533E-02	0,047738
Bt.2318.2.S1_at				1,294	2,452	1,580E-02	0,048890

Bt.22126.1.S1_at	TSPAN5	538668	tetraspanin 5	-1,380	-2,603	1,005E-02	0,037836
Bt.18202.1.A1_at				-1,456	-2,743	6,641E-03	0,032783
Bt.8319.1.S1_at	MTUS1	515016	mitochondrial tumor suppressor 1	-1,482	-2,794	5,714E-03	0,030409
Bt.1927.1.S1_at	CRISPLD2	505329	cysteine-rich secretory protein LCCL domain containing 2	-1,550	-2,928	3,855E-03	0,025555
Bt.2047.1.S1_at	ADM	280713	adrenomedullin	-1,816	-3,522	7,108E-04	0,009693
Bt.24314.1.A1_at				-1,953	-3,871	2,744E-04	0,007072
Bt.4209.2.S1_a_at	C3	280677	complement component 3	-1,983	-3,954	2,198E-04	0,006374
Bt.15290.1.A1_at				-2,209	-4,625	3,879E-05	0,002707

Probesets differentially expressed in 534 infected vs. control (cut-off: p.adj <= 0.05)

probeset ID	Gene symbol	Gene Entrez ID	Gene name	logFC	FC	P.Value	adj.P.Val
Bt.24940.1.A1_at				2,762	6,784	2,698E-06	1,043E-04
Bt.5395.1.S1_a_at	VCAN	282662	versican	2,116	4,336	3,182E-04	2,636E-03
Bt.24975.1.S1_at	YTHDC2	541024	YTH domain containing 2	2,037	4,104	5,290E-04	3,880E-03
Bt.20867.1.A1_at	RPUSD3	534606	RNA pseudouridylate synthase domain containing 3	1,988	3,966	7,210E-04	4,646E-03
Bt.7839.1.A1_at	SON	516462	SON DNA binding protein	1,978	3,939	7,658E-04	4,716E-03
Bt.6973.2.A1_at				1,976	3,935	7,725E-04	4,716E-03
Bt.21695.1.S1_at				1,962	3,897	8,410E-04	4,902E-03
Bt.25081.1.S1_at				1,957	3,881	8,718E-04	4,994E-03
Bt.20407.2.A1_at	CPEB4	538794	cytoplasmic polyadenylation element binding protein 4	1,933	3,818	1,007E-03	5,498E-03
Bt.12164.1.S1_at				1,909	3,754	1,164E-03	6,210E-03
Bt.8521.1.A1_at				1,890	3,707	1,298E-03	6,766E-03
Bt.27023.1.S1_at				1,868	3,649	1,483E-03	7,237E-03
Bt.2451.2.A1_at	CREBZF	538594	CREB/ATF bZIP transcription factor	1,861	3,632	1,543E-03	7,237E-03
Bt.8872.1.S1_at	ATP2B1	282641	ATPase, Ca++ transporting, plasma membrane 1	1,850	3,606	1,639E-03	7,237E-03
Bt.19425.1.S1_at	LARP4	539286	La ribonucleoprotein domain family, member 4	1,849	3,601	1,657E-03	7,237E-03
Bt.22714.1.A1_at	TMOD3	533257	tropomodulin 3 (ubiquitous)	1,848	3,601	1,658E-03	7,237E-03
Bt.3105.1.S1_at	CLINT1	538540	clathrin interactor 1	1,848	3,599	1,664E-03	7,237E-03
Bt.10920.1.S1_at	TTC3	506569	tetratricopeptide repeat domain 3	1,847	3,598	1,669E-03	7,237E-03
Bt.28265.2.S1_at				1,833	3,564	1,809E-03	7,609E-03
Bt.27865.2.S1_at	VPRBP		Vpr (HIV-1) binding protein	1,832	3,561	1,820E-03	7,609E-03
Bt.3741.1.S1_at	TSC1	533440	tuberous sclerosis 1	1,828	3,551	1,863E-03	7,718E-03
Bt.27429.2.A1_at	SF3B1	510177	splicing factor 3b, subunit 1, 155kDa	1,824	3,541	1,905E-03	7,823E-03
Bt.20759.1.A1_at				1,811	3,510	2,051E-03	8,130E-03
Bt.11220.2.S1_a_at	RALB	505238	v-ral simian leukemia viral oncogene homolog B (ras related; GTP binding protein)	1,810	3,507	2,068E-03	8,130E-03
Bt.4773.3.S1_a_at	ATP6V1A	282147	ATPase, H+ transporting, lysosomal 70kDa, V1 subunit A	1,797	3,476	2,223E-03	8,667E-03
Bt.12001.3.A1_at	ARFGEF2	539295	ADP-ribosylation factor guanine nucleotide-exchange factor 2 (brefeldin A-inhibited)	1,760	3,386	2,745E-03	1,043E-02

Bt.18234.1.S1_at	IGSF6	100140740	similar to DORA protein	1,758	3,383	2,766E-03	1,043E-02
Bt.10884.2.S1_at				1,753	3,370	2,853E-03	1,063E-02
Bt.22543.1.S1_at				1,750	3,364	2,893E-03	1,063E-02
Bt.16683.1.S1_at	C1D	506667	C1D nuclear receptor co-repressor	1,750	3,364	2,897E-03	1,063E-02
Bt.864.2.S1_a_at	SRSF1	615796	splicing factor, arginine/serine-rich 1	1,746	3,355	2,958E-03	1,072E-02
Bt.27936.1.S1_at				1,728	3,313	3,268E-03	1,149E-02
Bt.6497.1.S1_at	SMEK2	516489	SMEK homolog 2, suppressor of mek1 (Dictyostelium)	1,723	3,302	3,355E-03	1,170E-02
Bt.25965.2.S1_at	NCOR1	507663	nuclear receptor co-repressor 1	1,711	3,273	3,596E-03	1,218E-02
Bt.27024.1.A1_at	CCAR1	767587	cell division cycle and apoptosis regulator 1	1,708	3,267	3,652E-03	1,221E-02
Bt.24391.1.A1_at				1,698	3,245	3,850E-03	1,249E-02
Bt.27436.1.A1_at	INO80	505992	INO80 homolog (S. cerevisiae)	1,693	3,234	3,946E-03	1,272E-02
Bt.7236.1.S1_at	PIK3R1	282307	phosphoinositide-3-kinase, regulatory subunit 1 (alpha)	1,682	3,209	4,190E-03	1,341E-02
Bt.2415.2.S1_at	ID2	505025	inhibitor of DNA binding 2, dominant negative helix-loop-helix protein	1,679	3,201	4,275E-03	1,349E-02
Bt.8824.1.S1_at				1,674	3,192	4,371E-03	1,364E-02
Bt.28626.1.S1_at	LOC521363	521363	similar to GC-rich sequence DNA-binding factor (GCF) (Transcription factor 9) (TCF-9)	1,672	3,186	4,438E-03	1,364E-02
Bt.26597.1.S1_at				1,665	3,170	4,606E-03	1,397E-02
Bt.23673.1.A1_at				1,656	3,152	4,810E-03	1,440E-02
Bt.8690.1.S1_at	PLEKHB2	783000	pleckstrin homology domain containing, family B (evectins) member 2; similar to pleckstrin homology domain containing, family B (evectins) member 2	1,653	3,145	4,901E-03	1,448E-02
Bt.3311.1.S1_at				1,652	3,142	4,930E-03	1,448E-02
Bt.28780.2.A1_at	FGD4	505234	FYVE, RhoGEF and PH domain containing 4	1,649	3,137	4,993E-03	1,457E-02
Bt.17193.1.A1_at				1,646	3,129	5,087E-03	1,466E-02
Bt.11240.1.A1_at	TET2	538481	similar to CXXC finger 6	1,641	3,119	5,219E-03	1,486E-02
Bt.6392.1.S1_at	TRPM6	527597	transient receptor potential cation channel, subfamily M, member 6	1,632	3,099	5,473E-03	1,539E-02
Bt.10692.1.S1_at	LOC100299693	100299693	PRP38 pre-mRNA processing factor 38 (yeast) domain containing B	1,628	3,092	5,576E-03	1,549E-02
Bt.2943.1.A1_at	CTR9	534265	Ctr9, Paf1/RNA polymerase II complex component, homolog (S. cerevisiae)	1,624	3,082	5,713E-03	1,574E-02
Bt.24759.2.S1_at				1,623	3,080	5,732E-03	1,574E-02
Bt.4781.1.S1_at	ADAM10	282132	ADAM metallopeptidase domain 10	1,619	3,072	5,841E-03	1,594E-02
Bt.656.2.S1_at	CORO1C	515798	coronin, actin binding protein, 1C	1,604	3,041	6,314E-03	1,675E-02
Bt.19295.2.S1_at	APOLD1	538827	apolipoprotein L domain containing 1	1,604	3,040	6,317E-03	1,675E-02
Bt.21987.1.S1_at	PPIG	783720	peptidylprolyl isomerase G (cyclophilin G)	1,600	3,032	6,445E-03	1,697E-02
Bt.910.3.S1_at	UFM1	530547	ubiquitin-fold modifier 1	1,599	3,029	6,501E-03	1,697E-02
Bt.11772.1.A1_at	C18H16orf87	614339	chromosome 18 open reading frame, human C16orf87	1,596	3,023	6,587E-03	1,698E-02
Bt.25082.1.A1_at	MCOLN3	514345	mucolipin 3	1,592	3,015	6,723E-03	1,718E-02

Bt.9445.1.S1_at	HMGXB3	509419	HMG box domain containing 3	1,591	3,013	6,756E-03	1,718E-02
Bt.18794.1.S1_at				1,589	3,008	6,840E-03	1,718E-02
Bt.20506.1.S1_at				1,588	3,007	6,856E-03	1,718E-02
Bt.28222.1.S1_at	STRN3	516375	striatin, calmodulin binding protein 3	1,587	3,005	6,888E-03	1,718E-02
Bt.20852.1.A1_at	GNAS	281793	GNAS complex locus	1,580	2,990	7,149E-03	1,760E-02
Bt.18519.1.A1_at	RSBN1	508231	round spermatid basic protein 1	1,579	2,988	7,180E-03	1,760E-02
Bt.8263.1.A1_at				1,577	2,983	7,266E-03	1,760E-02
Bt.21412.1.A1_at	TXLNG	515927	taxilin gamma	1,576	2,981	7,310E-03	1,760E-02
Bt.179.1.S1_at	SLC25A16	282524	solute carrier family 25 (mitochondrial carrier; Graves disease autoantigen), member 16	1,575	2,980	7,319E-03	1,760E-02
Bt.18551.1.S1_at	PHF20L1	511990	PHD finger protein 20-like 1	1,570	2,970	7,516E-03	1,798E-02
Bt.11538.1.A1_at				1,546	2,919	8,510E-03	2,015E-02
Bt.10260.1.S1_at				1,537	2,902	8,884E-03	2,073E-02
Bt.27421.1.S1_at	ARHGEF2	505940	rho/rac guanine nucleotide exchange factor (GEF) 2	1,537	2,902	8,888E-03	2,073E-02
Bt.16140.1.A1_at	AGPAT5	530414	1-acylglycerol-3-phosphate O-acyltransferase 5 (lysophosphatidic acid acyltransferase, epsilon)	1,537	2,901	8,892E-03	2,073E-02
Bt.27858.1.A1_at	TMED5	534351	transmembrane emp24 protein transport domain containing 5	1,528	2,884	9,276E-03	2,141E-02
Bt.21444.1.S1_at	CDK2	519217	cyclin-dependent kinase 2	1,518	2,864	9,758E-03	2,230E-02
Bt.5928.1.S1_at	HMBOX1	539099	homeobox containing 1	1,515	2,859	9,890E-03	2,249E-02
Bt.18474.1.A1_at	SHOC2	511417	soc-2 suppressor of clear homolog (C. elegans)	1,513	2,854	1,000E-02	2,264E-02
Bt.829.2.S1_at	GOLPH3	531359	golgi phosphoprotein 3 (coat-protein)	1,502	2,833	1,055E-02	2,348E-02
Bt.3072.2.S1_at				1,500	2,829	1,065E-02	2,354E-02
Bt.4874.2.S1_a_at	LUC7L3	539955	cisplatin resistance-associated overexpressed protein	1,490	2,809	1,120E-02	2,460E-02
Bt.28462.1.S1_at	LTV1	507702	LTV1 homolog (S. cerevisiae)	1,489	2,807	1,124E-02	2,460E-02
Bt.8292.3.S1_at	MED13L	517336	mediator complex subunit 13-like	1,484	2,797	1,152E-02	2,510E-02
Bt.24703.1.S1_at	NBN	522943	nibrin	1,480	2,790	1,172E-02	2,541E-02
Bt.16148.2.A1_a_at	LOC532848	532848	centrosomal protein KIAA1731-like	1,477	2,783	1,193E-02	2,561E-02
Bt.6863.1.A1_at				1,472	2,775	1,218E-02	2,592E-02
Bt.13419.2.A1_at	SEC63	541040	SEC63 homolog (S. cerevisiae)	1,471	2,772	1,228E-02	2,601E-02
Bt.22440.1.S1_at	DCAF12	525161	WD repeat domain 40A	1,466	2,762	1,257E-02	2,652E-02
Bt.20414.3.S1_at	ROCK1	785911	E2F transcription factor 6; Rho-associated, coiled-coil containing protein kinase 2	1,464	2,760	1,266E-02	2,657E-02
Bt.21855.1.S1_at	TMED7	100125926	transmembrane emp24 protein transport domain containing 7	1,456	2,744	1,317E-02	2,728E-02
Bt.27028.1.S1_at				1,456	2,744	1,317E-02	2,728E-02
Bt.21170.1.A1_at	RASA2	533491	RAS p21 protein activator 2	1,444	2,721	1,395E-02	2,841E-02
Bt.22020.2.S1_at	LIN7C	510125	lin-7 homolog C (C. elegans)	1,444	2,720	1,397E-02	2,841E-02
Bt.22840.1.S1_a_at	CD46	280851	CD46 molecule, complement regulatory protein	1,437	2,707	1,443E-02	2,911E-02
Bt.15629.1.S1_at	NSRP1	539841	nuclear speckle splicing regulatory protein 1	1,434	2,702	1,464E-02	2,940E-02

Bt.18165.1.A1_at	MAPK6	538094	mitogen-activated protein kinase 6	1,432	2,698	1,476E-02	2,953E-02
Bt.4079.2.S1_a_at	TARDBP	540632	TAR DNA binding protein	1,431	2,696	1,484E-02	2,955E-02
Bt.2808.1.S1_at	RBM18	508615	RNA binding motif protein 18	1,422	2,679	1,548E-02	3,043E-02
Bt.13222.1.S1_at	TMX3	615687	thioredoxin-related transmembrane protein 3	1,419	2,674	1,570E-02	3,048E-02
Bt.16061.2.S1_at	COG6	509305	component of oligomeric golgi complex 6	1,417	2,670	1,587E-02	3,067E-02
Bt.20258.2.S1_at				1,409	2,655	1,646E-02	3,147E-02
Bt.633.2.S1_at	SFXN1	511375	sideroflexin 1	1,406	2,649	1,670E-02	3,164E-02
Bt.4823.1.S1_at	ARPP19	282658	cAMP-regulated phosphoprotein, 19 kD	1,405	2,649	1,673E-02	3,164E-02
Bt.28408.1.S1_at	MED1	520734	mediator complex subunit 1	1,404	2,646	1,684E-02	3,164E-02
Bt.20271.1.S1_at				1,404	2,646	1,684E-02	3,164E-02
Bt.29959.1.S1_at	CNOT6	534707	CCR4-NOT transcription complex, subunit 6	1,401	2,641	1,703E-02	3,187E-02
Bt.2891.1.S1_at	YWHAZ	287022	tyrosine 3-monooxygenase/tryptophan 5-monooxygenase activation protein, zeta polypeptide	1,396	2,632	1,743E-02	3,248E-02
Bt.3689.3.S1_a_at	C28H10orf35	510161	chromosome 10 open reading frame 35 ortholog	1,391	2,623	1,783E-02	3,310E-02
Bt.20939.1.A1_at				1,388	2,617	1,814E-02	3,352E-02
Bt.16142.3.A1_at	YWHAG	286862	tyrosine 3-monooxygenase/tryptophan 5-monooxygenase activation protein, gamma polypeptide	1,386	2,613	1,829E-02	3,367E-02
Bt.23985.1.A1_at	ATRX	514473	alpha thalassemia/mental retardation syndrome X-linked (RAD54 homolog, S. cerevisiae)	1,378	2,598	1,899E-02	3,482E-02
Bt.4554.1.S1_at	COMMD8	507292	COMM domain containing 8	1,377	2,597	1,907E-02	3,483E-02
Bt.4824.1.A1_at	EXOC5	531907	exocyst complex component 5	1,375	2,594	1,918E-02	3,490E-02
Bt.23224.2.S1_at				1,365	2,576	2,008E-02	3,580E-02
Bt.7092.1.S1_at				1,365	2,575	2,013E-02	3,580E-02
Bt.17473.1.S1_at	RPE	533764	ribulose-5-phosphate-3-epimerase	1,360	2,567	2,055E-02	3,639E-02
Bt.15742.1.S1_at	CD47	282661	CD47 molecule	1,354	2,556	2,113E-02	3,714E-02
Bt.19472.1.A1_at	FAM175B	510233	similar to LOC510233 protein; hypothetical protein LOC510233	1,352	2,552	2,134E-02	3,736E-02
Bt.7389.3.S1_a_at	TCERG1	540964	transcription elongation regulator 1	1,348	2,545	2,175E-02	3,794E-02
Bt.21372.1.S1_at	WHSC1L1	513054	Wolf-Hirschhorn syndrome candidate 1-like 1	1,345	2,541	2,199E-02	3,821E-02
Bt.24286.1.S1_at	GPR137B	613826	G protein-coupled receptor 137B	1,342	2,536	2,227E-02	3,854E-02
Bt.27072.1.A1_at	PB1	506557	polybromo 1	1,342	2,534	2,234E-02	3,854E-02
Bt.16374.1.A1_at	FUBP3	541080	far upstream element (FUSE) binding protein 3	1,341	2,533	2,244E-02	3,856E-02
Bt.4557.1.S1_at	IFNAR1	282257	interferon, alpha; receptor	1,339	2,530	2,262E-02	3,861E-02
Bt.807.1.S1_at				1,339	2,529	2,263E-02	3,861E-02
Bt.9300.1.A1_at	OGFRL1	519626	opioid growth factor receptor-like 1	1,337	2,527	2,277E-02	3,870E-02
Bt.9591.2.S1_at				1,336	2,525	2,291E-02	3,879E-02
Bt.7805.2.S1_a_at	NUCKS1	505585	nuclear casein kinase and cyclin-dependent kinase substrate 1; similar to Nuclear casein kinase and cyclin-dependent kinase substrate 1	1,335	2,523	2,300E-02	3,880E-02
Bt.12957.1.A1_a_at	TNRC6B	528167	trinucleotide repeat containing 6B	1,330	2,513	2,356E-02	3,937E-02

Bt.25022.1.S1_at				1,329	2,512	2,367E-02	3,937E-02
Bt.17161.1.S1_at				1,329	2,512	2,367E-02	3,937E-02
Bt.5602.1.A1_at	ZNF331	516156	hypothetical protein LOC516156	1,323	2,502	2,425E-02	4,004E-02
Bt.2763.1.S1_at	PARG	281377	poly (ADP-ribose) glycohydrolase	1,321	2,498	2,448E-02	4,028E-02
Bt.10033.1.S1_at	TNRC6A	541118	trinucleotide repeat containing 6A	1,317	2,492	2,487E-02	4,069E-02
Bt.16241.1.A1_at				1,315	2,489	2,510E-02	4,075E-02
Bt.22716.1.A1_at	RUFY1	528153	RUN and FYVE domain containing 1	1,315	2,488	2,512E-02	4,075E-02
Bt.23129.2.S1_at	LAMC1	532572	laminin, gamma 1 (formerly LAMB2)	1,312	2,483	2,548E-02	4,120E-02
Bt.26316.1.A1_at				1,309	2,478	2,581E-02	4,151E-02
Bt.3414.3.A1_a_at	AGFG1	536149	ArfGAP with FG repeats 1	1,307	2,474	2,604E-02	4,167E-02
Bt.6455.1.S1_at	CUL4A	617935	cullin 4A	1,305	2,470	2,629E-02	4,192E-02
Bt.9326.1.S1_at	DCUN1D1	539869	DCN1, defective in cullin neddylation 1, domain containing 1 (S. cerevisiae)	1,302	2,465	2,664E-02	4,216E-02
Bt.18224.1.S1_at				1,301	2,465	2,670E-02	4,216E-02
Bt.28468.1.A1_at	SMAD5	768050	SMAD family member 5	1,301	2,464	2,675E-02	4,216E-02
Bt.26811.1.S1_at	SLC30A5	508169	solute carrier family 30 (zinc transporter), member 5	1,300	2,462	2,689E-02	4,216E-02
Bt.21173.1.S1_at	HUWE1	511826	HECT, UBA and WWE domain containing 1	1,296	2,455	2,738E-02	4,267E-02
Bt.13455.2.A1_at	SACM1L	530577	SAC1 suppressor of actin mutations 1-like (yeast)	1,295	2,453	2,748E-02	4,267E-02
Bt.17129.1.A1_at				1,290	2,445	2,803E-02	4,321E-02
Bt.25433.1.S1_at	C1GALT1C1	531644	C1GALT1-specific chaperone 1	1,280	2,429	2,926E-02	4,480E-02
Bt.1781.1.A1_at				1,278	2,425	2,951E-02	4,504E-02
Bt.7610.1.A1_at				1,277	2,423	2,968E-02	4,515E-02
Bt.28376.1.S1_at				1,269	2,409	3,075E-02	4,663E-02
Bt.10397.2.S1_at	RNF20	513326	ring finger protein 20	1,263	2,400	3,150E-02	4,745E-02
Bt.4976.1.S1_at				1,260	2,396	3,183E-02	4,780E-02
Bt.13285.1.A1_at	LARP1	505690	La ribonucleoprotein domain family, member 1	1,260	2,394	3,195E-02	4,783E-02
Bt.22341.1.A1_at	VPS13C	783566	vacuolar protein sorting 13 homolog C (S. cerevisiae)	1,256	2,388	3,246E-02	4,842E-02
Bt.28267.2.S1_a_at				1,249	2,376	3,348E-02	4,925E-02
Bt.269.1.S1_at	ATP2C1	327663	ATPase, Ca++ transporting, type 2C, member 1	1,247	2,374	3,365E-02	4,925E-02
Bt.24333.1.S1_at	TLK1	537517	tousled-like kinase 1	1,247	2,374	3,369E-02	4,925E-02
Bt.23143.2.S1_at	CSDE1	513993	cold shock domain containing E1, RNA-binding	1,246	2,372	3,381E-02	4,925E-02
Bt.20762.2.S1_at	SPRYD7	615298	SPRY domain containing 7	1,246	2,372	3,386E-02	4,925E-02
Bt.2294.1.S1_a_at	UBA7	497204	ubiquitin-like modifier activating enzyme 7	-1,249	-2,376	3,345E-02	4,925E-02
Bt.100.1.S1_at	ADRB2	281605	adrenergic, beta-2-, receptor, surface	-1,288	-2,441	2,833E-02	4,353E-02
Bt.24231.1.A1_at	CEP78	537926	centrosomal protein 78kDa	-1,290	-2,446	2,798E-02	4,321E-02
Bt.8436.1.S1_at	IFI6	512913	interferon, alpha-inducible protein 6	-1,358	-2,563	2,078E-02	3,666E-02
Bt.9792.1.S1_at	ARRDC4	538517	arrestin domain containing 4	-1,411	-2,658	1,632E-02	3,143E-02
Bt.16725.2.A1_at	UBE2L6	509471	ubiquitin-conjugating enzyme E2L 6	-1,424	-2,683	1,535E-02	3,043E-02
Bt.26735.1.S1_at	SLC46A2	511556	solute carrier family 46, member 2	-1,476	-2,782	1,198E-02	2,561E-02
Bt.12327.1.S1_at	TXNIP	506790	thioredoxin interacting protein	-1,502	-2,831	1,058E-02	2,348E-02

Bt.22980.1.S1_at	TRIM21	359715	tripartite motif-containing 21	-1,811	-3,510	2,052E-03	8,130E-03
Bt.8962.3.S1_s_at	USP18	515202	ubiquitin specific peptidase 18	-1,854	-3,615	1,605E-03	7,237E-03
Bt.8238.1.A1_at	TRIB3	538465	tribbles homolog 3 (Drosophila)	-1,859	-3,627	1,561E-03	7,237E-03
Bt.16326.1.S1_at	TMEM140	515475	transmembrane protein 140	-2,082	-4,233	3,981E-04	3,078E-03
Bt.9519.1.S1_at	SESN2	509863	sestrin 2	-2,304	-4,939	8,914E-05	9,618E-04

Supplementary Material - S2 -

Gene Functional Classification

s303-control, 1 cluster

Gene Group 1	Enrichment Score: 0.5675		
**AFFYMETRIX_ID**	**Gene Name**	**Gene Symbol**	**Up/Down**
Bt.24697.1.A1_at	kinesin family member 3A	KIF3A	1
Bt.28345.1.S1_at	ankyrin repeat domain 17	ANKRD17	1
Bt.19645.1.A1_at	chromodomain helicase DNA binding protein 1	CHD1	1
Bt.4095.2.S1_at	torsin family 1, member B (torsin B)	TOR1B	1

Category	Term	Count	PValue
GOTERM_MF_FAT	GO:0005524~ATP binding	4	0,0013
GOTERM_MF_FAT	GO:0032559~adenyl ribonucleotide binding	4	0,0014
GOTERM_MF_FAT	GO:0030554~adenyl nucleotide binding	4	0,0016
GOTERM_MF_FAT	GO:0001883~purine nucleoside binding	4	0,0016
GOTERM_MF_FAT	GO:0001882~nucleoside binding	4	0,0017
GOTERM_MF_FAT	GO:0032555~purine ribonucleotide binding	4	0,0028
GOTERM_MF_FAT	GO:0032553~ribonucleotide binding	4	0,0028
GOTERM_MF_FAT	GO:0017076~purine nucleotide binding	4	0,0032
GOTERM_MF_FAT	GO:0000166~nucleotide binding	4	0,0053

s534-control, 7 clusters

Gene Group 1	Enrichment Score: 2.0353		
**AFFYMETRIX_ID**	**Gene Name**	**Gene Symbol**	**Up/Down**
Bt.2808.1.S1_at	RNA binding motif protein 18	RBM18	1
Bt.27023.1.S1_at	RNA-binding region (RNP1, RRM) containing 3	NA	1
Bt.4079.2.S1_a_at	TAR DNA binding protein	TARDBP	1
Bt.18224.1.S1_at	CUG triplet repeat, RNA binding protein 1		1
Bt.20407.2.A1_at	cytoplasmic polyadenylation element binding protein 4	CPEB4	1

Category	Term	Count	PValue
INTERPRO	IPR012677:Nucleotide-binding, alpha-beta plait	5	2,982E-08
INTERPRO	IPR000504:RNA recognition motif, RNP-1	5	3,185E-08
SMART	SM00360:RRM	5	6,216E-07
GOTERM_MF_FAT	GO:0000166~nucleotide binding	5	9,268E-04

SP_PIR_KEYWORDS	rna-binding	2	0,0241

**Gene Group 2**	Enrichment Score: 1.76603		
**AFFYMETRIX_ID**	**Gene Name**	**Gene Symbol**	**Up/Down**
Bt.2294.1.S1_a_at	ubiquitin-like modifier activating enzyme 7	UBA7	-1
Bt.16725.2.A1_at	ubiquitin-conjugating enzyme E2L 6	UBE2L6	-1
Bt.17148.2.S1_at	fem-1 homolog c (C. elegans)	FEM1C	1
Bt.8962.3.S1_s_at	ubiquitin specific peptidase 18	USP18	-1
Bt.8263.1.A1_at	hect (homologous to the E6-AP (UBE3A) carboxyl terminus) domain and RCC1 (CHC1)-like domain (RLD) 1		1
Bt.910.3.S1_at	ubiquitin-fold modifier 1	UFM1	1
Bt.6455.1.S1_at	cullin 4A	CUL4A	1

**Category**	**Term**	**Count**	**PValue**
GOTERM_BP_FAT	GO:0043632~modification-dependent macromolecule catabolic process	7	2,716E-10
GOTERM_BP_FAT	GO:0019941~modification-dependent protein catabolic process	7	2,716E-10
GOTERM_BP_FAT	GO:0051603~proteolysis involved in cellular protein catabolic process	7	4,655E-10
GOTERM_BP_FAT	GO:0044257~cellular protein catabolic process	7	4,770E-10
GOTERM_BP_FAT	GO:0030163~protein catabolic process	7	7,632E-10
GOTERM_BP_FAT	GO:0044265~cellular macromolecule catabolic process	7	1,230E-09
GOTERM_BP_FAT	GO:0009057~macromolecule catabolic process	7	2,544E-09
GOTERM_BP_FAT	GO:0006508~proteolysis	7	1,151E-07
GOTERM_BP_FAT	GO:0032020~ISG15-protein conjugation	3	2,331E-06
KEGG_PATHWAY	bta04120:Ubiquitin mediated proteolysis	4	1,953E-05
SP_PIR_KEYWORDS	ubl conjugation pathway	4	5,129E-05

**Gene Group 3**	Enrichment Score: 1.6820		
**AFFYMETRIX_ID**	**Gene Name**	**Gene Symbol**	**Up/Down**
Bt.21170.1.A1_at	RAS p21 protein activator 2	RASA2	1
Bt.21982.1.S1_a_at	IQ motif containing GTPase activating protein 1	IQGAP1	1
Bt.24678.1.A1_at	TBC1 domain family, member 9 (with GRAM domain)	TBC1D9	1
Bt.20947.2.S1_at	similar to IQ motif containing GTPase activating protein 2	LOC100140708	1

Bt.28780.2.A1_at	FYVE, RhoGEF and PH domain containing 4	FGD4	1

Category	Term	Count	PValue
GOTERM_BP_FAT	GO:0051056~regulation of small GTPase mediated signal transduction	5	1,143E-07
GOTERM_MF_FAT	GO:0030695~GTPase regulator activity	5	2,584E-07
GOTERM_MF_FAT	GO:0060589~nucleoside-triphosphatase regulator activity	5	2,949E-07
GOTERM_MF_FAT	GO:0005096~GTPase activator activity	4	4,037E-06
GOTERM_MF_FAT	GO:0008047~enzyme activator activity	4	1,099E-05
GOTERM_MF_FAT	GO:0005083~small GTPase regulator activity	4	1,524E-05
GOTERM_MF_FAT	GO:0005099~Ras GTPase activator activity	3	1,319E-04
GOTERM_BP_FAT	GO:0046578~regulation of Ras protein signal transduction	2	0,0562
KEGG_PATHWAY	bta04810:Regulation of actin cytoskeleton	2	0,0755

Gene Group 4	Enrichment Score: 1.3822		
**AFFYMETRIX_ID**	**Gene Name**	**Gene Symbol**	**Up/Down**
Bt.18165.1.A1_at	mitogen-activated protein kinase 6	MAPK6	1
Bt.9710.1.S1_at	PRP4 pre-mRNA processing factor 4 homolog B (yeast)	PRPF4B	1
Bt.27436.1.A1_at	INO80 homolog (S. cerevisiae)	INO80	1
Bt.8238.1.A1_at	tribbles homolog 3 (Drosophila)	TRIB3	-1
Bt.21444.1.S1_at	cyclin-dependent kinase 2	CDK2	1
Bt.24975.1.S1_at	YTH domain containing 2	YTHDC2	1
Bt.4095.2.S1_at	torsin family 1, member B (torsin B)	TOR1B	1
Bt.6392.1.S1_at	transient receptor potential cation channel, subfamily M, member 6	TRPM6	1
Bt.16725.2.A1_at	ubiquitin-conjugating enzyme E2L 6	UBE2L6	-1
Bt.24697.1.A1_at	kinesin family member 3A	KIF3A	1
Bt.19645.1.A1_at	chromodomain helicase DNA binding protein 1	CHD1	1
Bt.2294.1.S1_a_at	ubiquitin-like modifier activating enzyme 7	UBA7	-1
Bt.23985.1.A1_at	alpha thalassemia/mental retardation syndrome X-linked (RAD54 homolog, S. cerevisiae)	ATRX	1
Bt.24333.1.S1_at	tousled-like kinase 1	TLK1	1
Bt.120.1.S1_at, Bt.20414.3.S1_at	E2F transcription factor 6; Rho-associated, coiled-coil containing protein kinase 2	ROCK2, ROCK1	1
Bt.24692.1.S1_at	adrenergic, beta, receptor kinase 2	ADRBK2	1

Category	Term	Count	PValue
GOTERM_MF_FAT	GO:0005524~ATP binding	16	3,843E-15
GOTERM_MF_FAT	GO:0032559~adenyl ribonucleotide binding	16	4,390E-15
GOTERM_MF_FAT	GO:0030554~adenyl nucleotide binding	16	9,787E-15
GOTERM_MF_FAT	GO:0001883~purine nucleoside binding	16	1,124E-14
GOTERM_MF_FAT	GO:0001882~nucleoside binding	16	1,221E-14
GOTERM_MF_FAT	GO:0032555~purine ribonucleotide binding	16	1,613E-13
GOTERM_MF_FAT	GO:0032553~ribonucleotide binding	16	1,613E-13
GOTERM_MF_FAT	GO:0017076~purine nucleotide binding	16	3,143E-13
GOTERM_MF_FAT	GO:0000166~nucleotide binding	16	4,053E-12
INTERPRO	IPR017442:Serine/threonine protein kinase-related	6	1,353E-07
UP_SEQ_FEATURE	domain:Protein kinase	5	2,745E-07
INTERPRO	IPR000719:Protein kinase, core	6	4,010E-07
GOTERM_BP_FAT	GO:0006468~protein amino acid phosphorylation	8	5,427E-07
SP_PIR_KEYWORDS	atp-binding	7	9,739E-07
INTERPRO	IPR002290:Serine/threonine protein kinase	5	1,421E-06
GOTERM_BP_FAT	GO:0016310~phosphorylation	8	1,513E-06
GOTERM_MF_FAT	GO:0004672~protein kinase activity	8	1,863E-06
GOTERM_MF_FAT	GO:0004674~protein serine/threonine kinase activity	7	2,902E-06
INTERPRO	IPR008271:Serine/threonine protein kinase, active site	5	5,348E-06
SP_PIR_KEYWORDS	serine/threonine-protein kinase	5	5,565E-06
SMART	SM00220:S_TKc	5	5,931E-06
GOTERM_BP_FAT	GO:0006796~phosphate metabolic process	8	6,433E-06
GOTERM_BP_FAT	GO:0006793~phosphorus metabolic process	8	6,433E-06
SP_PIR_KEYWORDS	nucleotide-binding	7	7,320E-06
INTERPRO	IPR017441:Protein kinase, ATP binding site	5	1,009E-05
UP_SEQ_FEATURE	binding site:ATP	4	7,036E-05
UP_SEQ_FEATURE	active site:Proton acceptor	4	3,703E-04
GOTERM_MF_FAT	GO:0004386~helicase activity	4	4,234E-04
UP_SEQ_FEATURE	nucleotide phosphate-binding region:ATP	4	5,392E-04
SP_PIR_KEYWORDS	kinase	4	0,0015
GOTERM_CC_FAT	GO:0043232~intracellular non-membrane-bounded organelle	5	0,0032
GOTERM_CC_FAT	GO:0043228~non-membrane-bounded organelle	5	0,0032
GOTERM_BP_FAT	GO:0032020~ISG15-protein conjugation	2	0,0055
SP_PIR_KEYWORDS	serine/threonine-specific protein kinase	2	0,0114
GOTERM_CC_FAT	GO:0005694~chromosome	3	0,0120

SP_PIR_KEYWORDS	transferase	4	0,0126
UP_SEQ_FEATURE	domain:AGC-kinase C-terminal	2	0,0132
INTERPRO	IPR000961:AGC-kinase, C-terminal	2	0,0199
SP_PIR_KEYWORDS	ATP	2	0,0254
SMART	SM00133:S_TK_X	2	0,0312
UP_SEQ_FEATURE	domain:PH	2	0,0324
SP_PIR_KEYWORDS	phosphotransferase	2	0,0329

Gene Group 5 Enrichment Score: 0.77695

AFFYMETRIX_ID	Gene Name	Gene Symbol	Up/Down
Bt.269.1.S1_at	ATPase, Ca++ transporting, type 2C, member 1	ATP2C1	1
Bt.4773.3.S1_a_at	ATPase, H+ transporting, lysosomal 70kDa, V1 subunit A	ATP6V1A	1
Bt.8872.1.S1_at	ATPase, Ca++ transporting, plasma membrane 1	ATP2B1	1
Bt.17311.2.S1_at	retinitis pigmentosa 2 (X-linked recessive)	RP2	1

Category	Term	Count	PValue
GOTERM_BP_FAT	GO:0009145~purine nucleoside triphosphate biosynthetic process	4	1,005E-06
GOTERM_BP_FAT	GO:0009206~purine ribonucleoside triphosphate biosynthetic process	4	1,005E-06
GOTERM_BP_FAT	GO:0009201~ribonucleoside triphosphate biosynthetic process	4	1,005E-06
GOTERM_BP_FAT	GO:0009142~nucleoside triphosphate biosynthetic process	4	1,040E-06
GOTERM_BP_FAT	GO:0009205~purine ribonucleoside triphosphate metabolic process	4	1,305E-06
GOTERM_BP_FAT	GO:0009199~ribonucleoside triphosphate metabolic process	4	1,305E-06
GOTERM_BP_FAT	GO:0009144~purine nucleoside triphosphate metabolic process	4	1,346E-06
GOTERM_BP_FAT	GO:0009152~purine ribonucleotide biosynthetic process	4	1,520E-06
GOTERM_BP_FAT	GO:0009141~nucleoside triphosphate metabolic process	4	1,757E-06
GOTERM_BP_FAT	GO:0009260~ribonucleotide biosynthetic process	4	1,757E-06
GOTERM_BP_FAT	GO:0009150~purine ribonucleotide metabolic process	4	2,073E-06

GOTERM_BP_FAT	GO:0006164~purine nucleotide biosynthetic process	4	2,303E-06
GOTERM_BP_FAT	GO:0009259~ribonucleotide metabolic process	4	2,363E-06
GOTERM_BP_FAT	GO:0006163~purine nucleotide metabolic process	4	3,393E-06
GOTERM_BP_FAT	GO:0009165~nucleotide biosynthetic process	4	4,880E-06
GOTERM_BP_FAT	GO:0034654~nucleobase, nucleoside, nucleotide and nucleic acid biosynthetic process	4	5,601E-06
GOTERM_BP_FAT	GO:0034404~nucleobase, nucleoside and nucleotide biosynthetic process	4	5,601E-06
GOTERM_BP_FAT	GO:0044271~nitrogen compound biosynthetic process	4	2,091E-05
GOTERM_MF_FAT	GO:0019829~cation-transporting ATPase activity	3	2,458E-05
GOTERM_MF_FAT	GO:0042625~ATPase activity, coupled to transmembrane movement of ions	3	1,236E-04
GOTERM_BP_FAT	GO:0006754~ATP biosynthetic process	3	2,504E-04
GOTERM_MF_FAT	GO:0042626~ATPase activity, coupled to transmembrane movement of substances	3	2,917E-04
GOTERM_BP_FAT	GO:0046034~ATP metabolic process	3	2,957E-04
GOTERM_MF_FAT	GO:0016820~hydrolase activity, acting on acid anhydrides, catalyzing transmembrane movement of substances	3	2,981E-04
GOTERM_MF_FAT	GO:0043492~ATPase activity, coupled to movement of substances	3	2,981E-04
GOTERM_MF_FAT	GO:0015405~P-P-bond-hydrolysis-driven transmembrane transporter activity	3	3,517E-04
GOTERM_MF_FAT	GO:0015399~primary active transmembrane transporter activity	3	3,517E-04
GOTERM_MF_FAT	GO:0022890~inorganic cation transmembrane transporter activity	3	3,874E-04
GOTERM_MF_FAT	GO:0042623~ATPase activity, coupled	3	0,0011
GOTERM_MF_FAT	GO:0005524~ATP binding	4	0,0013
GOTERM_MF_FAT	GO:0032559~adenyl ribonucleotide binding	4	0,0014
GOTERM_MF_FAT	GO:0016887~ATPase activity	3	0,0016
GOTERM_MF_FAT	GO:0030554~adenyl nucleotide binding	4	0,0016
GOTERM_MF_FAT	GO:0001883~purine nucleoside binding	4	0,0016
GOTERM_MF_FAT	GO:0001882~nucleoside binding	4	0,0017
INTERPRO	IPR004014:ATPase, P-type cation-transporter, N-terminal	2	0,0020

INTERPRO	IPR006068:ATPase, P-type cation-transporter, C-terminal	2	0,0023
GOTERM_MF_FAT	GO:0015085~calcium ion transmembrane transporter activity	2	0,0026
GOTERM_MF_FAT	GO:0005388~calcium-transporting ATPase activity	2	0,0026
INTERPRO	IPR018303:ATPase, P-type phosphorylation site	2	0,0026
GOTERM_MF_FAT	GO:0032555~purine ribonucleotide binding	4	0,0028
GOTERM_MF_FAT	GO:0032553~ribonucleotide binding	4	0,0028
INTERPRO	IPR008250:ATPase, P-type, ATPase-associated region	2	0,0029
PIR_SUPERFAMILY	PIRSF001297:Na(+)/K(+)-transporting ATPase alpha chain	2	0,0031
GOTERM_MF_FAT	GO:0017076~purine nucleotide binding	4	0,0032
INTERPRO	IPR001757:ATPase, P-type, K/Mg/Cd/Cu/Zn/Na/Ca/Na/H-transporter	2	0,0042
GOTERM_BP_FAT	GO:0006812~cation transport	3	0,0048
GOTERM_MF_FAT	GO:0000166~nucleotide binding	4	0,0053
SP_PIR_KEYWORDS	atp-binding	3	0,0053
GOTERM_MF_FAT	GO:0015082~di-, tri-valent inorganic cation transmembrane transporter activity	2	0,0062
INTERPRO	IPR005834:Haloacid dehalogenase-like hydrolase	2	0,0069
SP_PIR_KEYWORDS	hydrolase	3	0,0088
GOTERM_BP_FAT	GO:0006811~ion transport	3	0,0097
SP_PIR_KEYWORDS	nucleotide-binding	3	0,0105
GOTERM_MF_FAT	GO:0015662~ATPase activity, coupled to transmembrane movement of ions, phosphorylative mechanism	2	0,0142
GOTERM_BP_FAT	GO:0006816~calcium ion transport	2	0,0254
GOTERM_BP_FAT	GO:0015674~di-, tri-valent inorganic cation transport	2	0,0351

**Gene Group 6**	Enrichment Score: 0.3898		
**AFFYMETRIX_ID**	**Gene Name**	**Gene Symbol**	**Up/Down**
Bt.21855.1.S1_at	transmembrane emp24 protein transport domain containing 7	TMED7	1
Bt.27858.1.A1_at	transmembrane emp24 protein transport domain containing 5	TMED5	1
Bt.22126.1.S1_at	tetraspanin 5	TSPAN5	-1
Bt.25433.1.S1_at	C1GALT1-specific chaperone 1	C1GALT1C1	1

Category	Term	Count	PValue
INTERPRO	IPR000348:emp24/gp25L/p24	2	0,0015
INTERPRO	IPR009038:GOLD	2	0,0020
SP_PIR_KEYWORDS	transmembrane	4	0,0111
UP_SEQ_FEATURE	topological domain:Cytoplasmic	3	0,0172
SP_PIR_KEYWORDS	membrane	4	0,0190
UP_SEQ_FEATURE	transmembrane region	3	0,0480

**Gene Group 7**	Enrichment Score: 0.0171		
**AFFYMETRIX_ID**	**Gene Name**	**Gene Symbol**	**Up/Down**
Bt.13990.2.S1_at	zinc finger, CCHC domain containing 8	ZCCHC8	1
Bt.23985.1.A1_at	alpha thalassemia/mental retardation syndrome X-linked (RAD54 homolog, S. cerevisiae)	ATRX	1
Bt.24537.1.A1_at	zinc finger, RAN-binding domain containing 2	ZRANB2	1
Bt.28780.2.A1_at	FYVE, RhoGEF and PH domain containing 4	FGD4	1

Category	Term	Count	PValue
GOTERM_MF_FAT	GO:0008270~zinc ion binding	4	0,0032
GOTERM_MF_FAT	GO:0046914~transition metal ion binding	4	0,0062
GOTERM_MF_FAT	GO:0046872~metal ion binding	4	0,0172
GOTERM_MF_FAT	GO:0043169~cation binding	4	0,0178
GOTERM_MF_FAT	GO:0043167~ion binding	4	0,0183
